# Abnormal lineage differentiation of peri‐implantation aneuploid embryos revealed by single‐cell RNA sequencing

**DOI:** 10.1002/ctm2.70326

**Published:** 2025-05-07

**Authors:** Xueyao Chen, Hanwen Yu, Yu Yin, Bing Cai, Gaohui shi, Yan Xu, Lujuan Rong, Xiu Yu, Boyan Wang, Canquan Zhou, Jichang Wang, Chenhui Ding, Tianqing Li, Yanwen Xu

**Affiliations:** ^1^ Department of Gynecology & Obstetrics, Center for Reproductive Medicine, The First Affiliated Hospital Sun Yat‐Sen University Guangzhou Guangdong China; ^2^ Guangdong Provincial Key Laboratory of Reproductive Medicine Guangzhou Guangdong China; ^3^ Guangdong Provincial Clinical Research Center for Obstetrical and Gynecological Diseases Guangzhou Guangdong China; ^4^ Advanced Medical Technology Center, The First Affiliated Hospital, Zhongshan School of Medicine Sun Yat‐Sen University Guangzhou Guangdong China; ^5^ Key Laboratory for Stem Cells and Tissue Engineering (Sun Yat‐Sen University), Ministry of Education Guangzhou Guangdong China; ^6^ Department of Histology and Embryology, Zhongshan School of Medicine Sun Yat‐Sen University Guangzhou Guangdong China; ^7^ Yunnan Key Laboratory of Primate Biomedical Research, Institute of Primate Translational Medicine Kunming University of Science and Technology Kunming Yunnan China

1

Dear Editor,

Early pregnancy loss is often caused by embryonic aneuploidy.^1^, ^2^ However, the developmental process of aneuploid embryos remains largely unexplored. In this study, we delineated the developmental pattern of aneuploid embryos at the peri‐implantation stage through 3D in vitro culture. A gain of chromosome 16 caused the premature development of trophoblasts, while a loss of chromosome 16 led to a blockage in trophoblast differentiation. We found that the *CREBBP* gene, located on the chr16, regulates the aberrant trophoblast development of monosomy 16 (M16) and trisomy 16 (T16) through a dosage effect, which was further validated in blastoids and TSCs (trophoblast stem cells) models. These findings provide insights into exploration of embryonic defects leading to repeated implantation failure or pregnancy loss.

Donated blastocysts were cultured until 12 days post‐fertilization (d.p.f. 12) by an in vitro 3D system established by Xiang et al.^3^ (Figure 1A). In euploid embryos, three embryonic lineages could be identified by specific lineage markers (Figure 1B, Note S1). Aneuploid embryos were significantly smaller in size and exhibited delayed development (Figure 1C). M16 embryos were significantly more likely to arrest before d.p.f.10 (52.6%, *n* = 10/19) than euploid embryos were (13.6%, *n* = 3/22, *p *= .017, Figure 1D). By immunofluorescence, aneuploid embryos exhibited abnormal morphology and poor epiblast (EPI) development (Figure 1E,F). In particular, M16 exhibited the lowest proportion of EPI cells, with only five EPI cells identified across all M16 embryos (*n* = 11). Few hCGB (+) STB cells (syncytiotrophoblast) were observed in the monosomy embryos, indicating that cell differentiation into STBs was restricted (Figure S1a and 3D,E).

**FIGURE 1:**
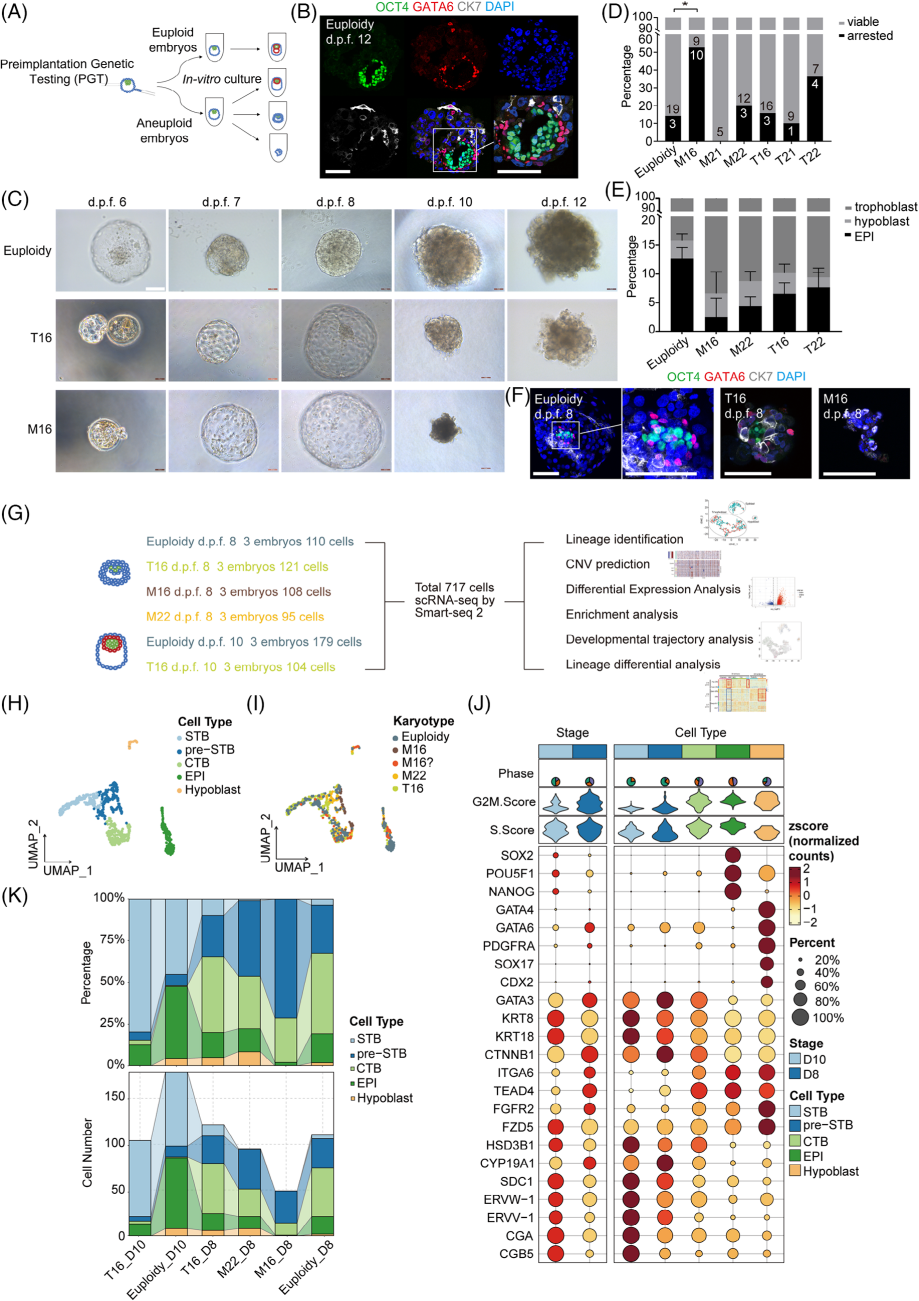
Peri‐implantation development of euploid and aneuploid human embryos. (A) Schematic representation of in vitro 3D culture for human blastocysts diagnosed by preimplantation genetic testing (PGT). (B) Immunostaining of d.p.f. 12 euploid embryos from in vitro culture. Specific markers identified different lineage cells: OCT4 for epiblasts, GATA6 for hypoblasts and CK7 for trophoblasts. Scale bar, 100 µm. (c) Sequential images from d.p.f. 6–12. images of euploid, T16 and M16 embryos in a 3D in vitro culture system. M16 embryos arrested after d.p.f. 8, failing to progress to d.p.f. 12. (D) The proportion of arrested and viable embryos at d.p.f. 10 in each karyotype. The number of embryos was indicated in the column. *p < .05. (E) The proportion of cells across three lineages in each karyotype. Cell lineage was determined by PCR or immunofluorescence. Specific markers identified different lineage cells: OCT4 or NANOG for epiblasts, GATA6 or PDGFRA for hypoblasts and CK7 or GATA3 for trophoblasts. *N* = 7 euploidy, 4 M21, 5 M22, 6 T16 and 5 T22 embryos. Error bars indicate ± SD. (F) Immunofluorescence of d.p.f. 8 embryos. Euploid embryo at d.p.f. 8 with normal morphology, possessing distinct epiblast cell clusters, with hypoblast cells enveloping the EPI cells. Aneuploid embryos at d.p.f. 8 with abnormal morphology and poor EPI development. Scale bar, 100 µm. (G) Schematic illustration of single‐cell collection and transcriptome analyses. (H) UMAP analyses revealed five clusters, identified as EPIs (epiblasts), hypoblasts and TrBs (trophoblasts, including CTBs (cytotrophoblast), pre‐STBs and STBs (syncytiotrophoblast)). A total of 717 cells derived from 18 embryos were included in the final analysis. (I) UMAP scatter plot grouped by karyotype. ‘M16?’ refers to an embryo which was identified as M16 by PGT karyotyping but was predicted to be euploid after in vitro culture based on CNV analysis. Detailed information is provided in Note S2. (J) Dot plot revealed the expression of lineage markers in each cluster. (K) Cell proportion and number of each lineage identified by scRNA‐seq across embryos of various karyotypes.

ScRNA‐seq analysis of 18 embryos at d.p.f. 8–10 was conducted for characterization of peri‐implantation embryo development (Figure 1G, Table S1). Following quality control (Figure S1b), 717 single cells with 40,874 genes (including non‐coding genes) were used for subsequent analyses. Seurat was employed for dimensionality reduction and unsupervised clustering analysis. Single cells were annotated into five cell types corresponding to lineage marker expression features (Figure 1H–J, Figure S1c). The UMAP plot showed that M16 embryos contained only trophoblast cells with abnormal distribution (Figure 1I). The EPI cell count was lower in aneuploid embryos than in euploid embryos, with M16 embryos exhibiting the fewest EPI cells (Figure 1K). Regarding the trophoblast lineage, T16 embryos contained more STB cells than euploid embryos, whereas M16 embryos had nearly none. M16 and M22 embryos had a greater abundance of pre‐STB cells (Figure 1K).

For global transcriptomic analysis, M16 and M22 embryos showed a halved copy number variation (CNV) on Chr 16 or 22, while T16 embryos showed a 1.5‐fold CNV increase on Chr 16 (Figure 2A); their inferred CNV levels predicted by R package inferCNV were consistent with the dosage levels of the aneuploid chromosomes (Note S2). Subsequently, we explored the transcriptional characteristics of the whole aneuploid embryos (Table S2 and S3). M16 embryos showed upregulation of cell death‐related pathways, including endocytosis, ferroptosis, FoxO signalling and FcγR‐mediated phagocytosis pathways. The downregulated genes were enriched in biological metabolism (Figure 2B,C). These findings suggested that M16 embryos had reduced metabolic capacity and initiated apoptosis at the peri‐implantation stage. T16 embryos at d.p.f. 10, showed upregulation of pathways related to protein processing, hormone synthesis and steroidogenesis (Figure 2D,E). GSEA analysis (Gene Set Enrichment Analysis) identified differential pathway enrichment patterns among distinct karyotypes (Figure 2F, Note S3). DEG distribution across chromosomes showed that the highest number of DEGs in aneuploid embryos was located on the aneuploid chromosomes, suggesting that the dosage effect predominantly affected the transcriptome (Figure 2G, Note S4).

**FIGURE 2:**
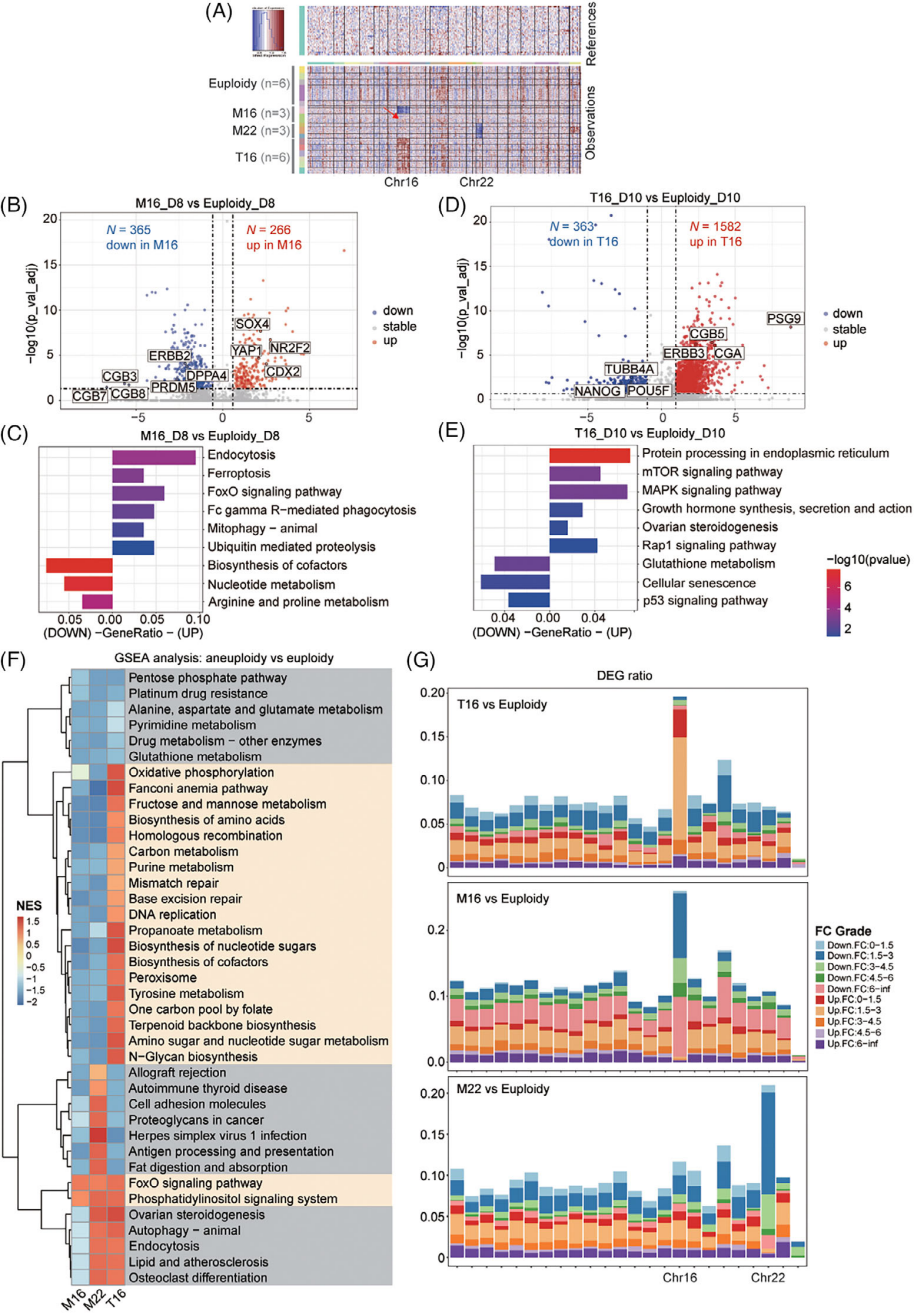
Global transcriptional characteristics of aneuploid embryos. (A) Prediction of embryo karyotype based on CNV analysis. The vertical axis of the heatmap represents the PGT diagnosis karyotype. The genes on the horizontal axis were arranged in chromosomal order. Chr16 and Chr22 of interest were marked. The concordance rate between the predicted karyotype and the PGT diagnosis was 94.44% (*n* = 17/18), with only one embryo's karyotype unmatched (red arrow). Sample sizes are indicated in the figure. (B, D) Volcano maps showing DEGs between euploid embryos and M16 embryos at d.p.f. 8 (B) or T16 embryos at d.p.f. 10 (D). (C) KEGG enrichment analysis revealed the dysregulated pathways in M16 embryos at d.p.f. 8. Pathways relevant to development, cell growth and differentiation are presented. (E) KEGG enrichment analysis showing the dysregulated pathways in T16 embryos at d.p.f. 10. (F) Heatmap summarizing significantly upregulated (orange) or downregulated (blue) pathways as determined by GSEA. The presented pathways were significantly altered (*p* < .05) in two or more karyotype comparisons. The value used for generating the heatmap were the NES (normal enrichment scores) from GSEA. NES reflects the strength and direction of a gene set's enrichment in ranked gene expression data (positive = upregulated, negative = downregulated). (G) Bar plot described the DEGs distribution across chromosomes. The DEG ratio is calculated by dividing the number of DEGs by the total number of genes on each chromosome, with colour coding based on the fold change.

Trophoblast development significantly influenced embryo implantation. To explore trophoblast development in aneuploid embryos, we extracted all trophoblast cells from the scRNA dataset for analysis. RNA velocity‐based developmental trajectory analysis suggested that d.p.f. 10 T16 trophoblast cells were more differentiated, whereas M16 trophoblast cells remained undifferentiated (Figure 3A). An increased proportion of cells in the G2/M phase was observed within M16 trophoblasts. Conversely, a greater proportion of T16 trophoblast cells in the G1 phase indicated reduced proliferation (Figure 3B). Next, we examined the expression profiles of specific gene sets highly relevant to trophoblast subtype differentiation^3^ (Figure 3C). In M16 embryos, CTB (cytotrophoblast)‐related genes (including *CDX2, NR2F2, SOX4* and *TFCP2L*) were upregulated, while STB‐related genes (including *CGA, CGB, PSG and ERVV*) and EVT (extravillous trophoblast)‐related genes (including *HLA‐G, ITGA1, DLX6* and *PRDM5*) were downregulated (Figure 3C, Figure S3a,b, Table S4). The expression of STB‐related genes generally increased at d.p.f. 8–10 in both euploid and aneuploid trophoblasts, indicating that physiological STB differentiation occurred during this period. Under this condition, STB‐related genes were upregulated in d.p.f. 8 T16 trophoblasts, and the upregulation became more prominent in d.p.f. 10 T16 trophoblasts (Figure 3C). The expression of STB regulatory genes (*TBX3, CREB1* and *SDC1*) did not significantly differ at 8 d.p.f. but increased significantly in T16 embryos at 10 d.p.f. (Figure S3c). By immunofluorescence, we determined that T16 embryos expressed significantly higher HCGB at 8–10 d.p.f. than euploid embryos (Figure 3D,E). Conversely, HCGB (+) cells were barely detected in M16 embryos. The Wnt signalling pathway was predominantly downregulated in T16 trophoblast but upregulated in M16 CTB and pre‐STB cells (Figure S4, Note S5, Table S5). Integrating our scRNA‐seq data with the published dataset,^3^ we validated that the trophoblast of the M16 embryo was in a stage of differentiation block, whereas the trophoblast of the T16 embryo prematurely over‐differentiated into the STB lineage (Figure 3F, Figure S3d, Note S6).

**FIGURE 3:**
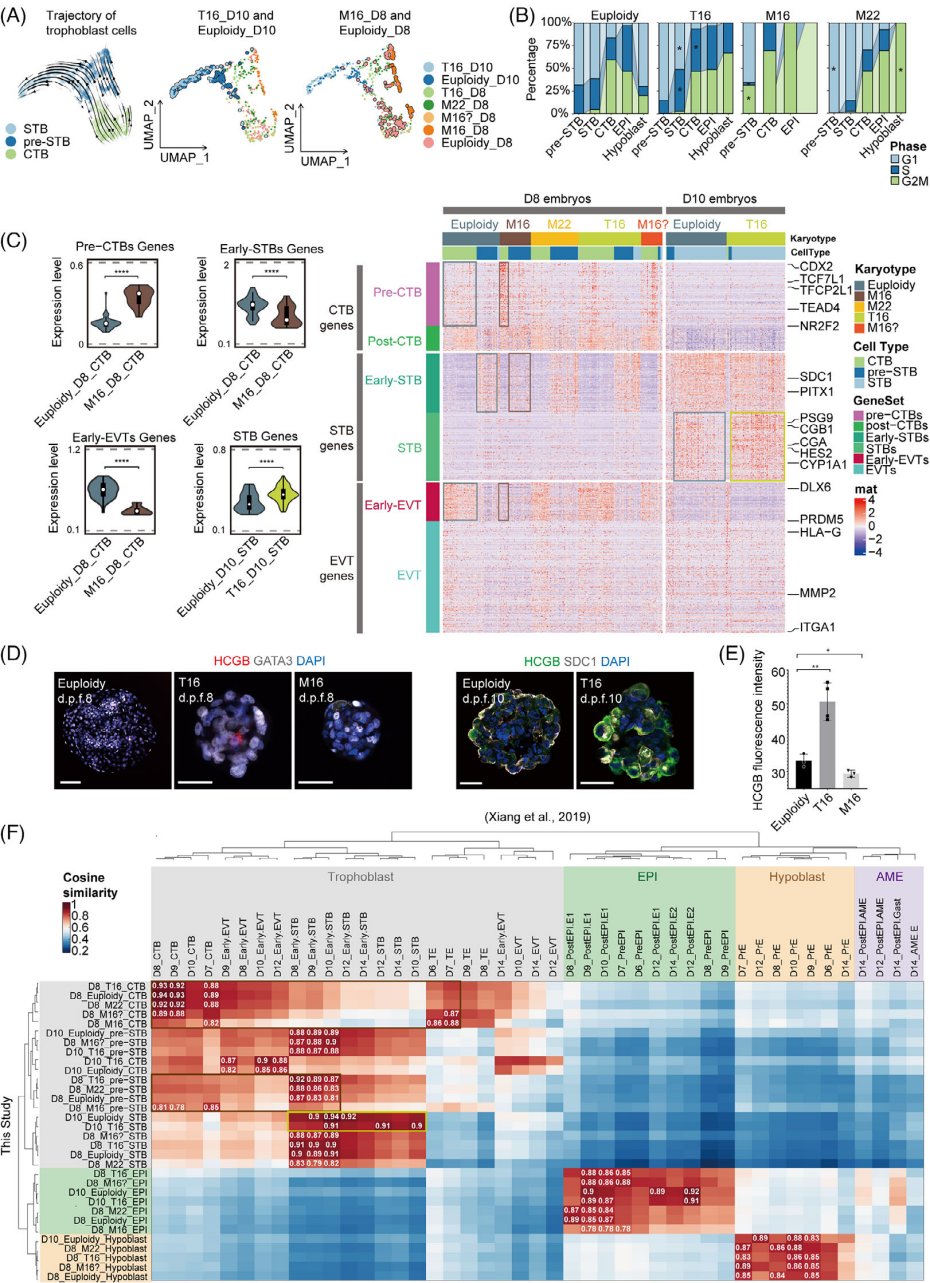
Abnormalities in trophoblast differentiation in aneuploid embryos. (A) The left panel: Trajectory analysis of the scRNA‐seq data from trophoblast cells of embryos at d.p.f. 8–10. Middle and left panels: UMAP dot plot of trophoblast cells, comparing the distributions of T16, M16 and euploid trophoblasts. Compared with euploid trophoblast cells, d.p.f. 10 T16 trophoblast cells were more differentiated, while M16 trophoblast cells remained undifferentiated. (B) Cell cycle analysis suggested that M16 trophoblasts had an increased proportion of cells in the G2/M phase, while T16 trophoblasts had a greater proportion of cells in the G1 phase. *p < .05, chi‐square test. (C) The expression patterns of genes specific to trophoblast subtypes among trophoblasts with diverse embryo karyotypes. The frames on the heatmap emphasized notable differences in expression profiles, with associated significance comparisons shown in the violin plots. The frame colour corresponds to the specific karyotypes detailed in the legend. ****p < .00005. (D) Immunofluorescence analysis revealed HCGB expression in the embryos at d.p.f. 8–10. Scale bar, 50 µm. (E) HCGB fluorescence intensity was significantly greater in T16 embryo trophoblasts than in euploid embryo trophoblasts. *N* = 3 euploidy, 4 T16 and 3 M16 embryos. *p < .05, **p < .005. (F) The heatmap depicts the correlation of various cell types and stages between this study's data and Xiang et al.’s data, with the numbers in the heatmap indicating the correlation coefficients of the top 3 groups with the highest relevance in each cell type.

To identify key genes regulating lineage differentiation in Chr16 aneuploid embryos, we screened for DEGs that met three criteria: upregulated in T16 STB, downregulated in M16 CTB or pre‐STB and located on Chr16. We identified 43 genes including *AARS1*, *APOBR*, *CREBBP*, *GAN*, etc. (Figure 4A, Table S6). Among them, *CREBBP* has been reported to be associated with trophoblast development.^4^, ^5^ CREBBP shares structural and functional similarities with EP300 and KAT8, both of which were reported to be involved in trophoblast differentiation.^4^, ^6^, ^7^ SCENIC‐based TF regulon analysis identified ELF3, CEBPA, CEBPB and FOXO1 as the most transcriptionally active regulators in aneuploid embryos (Figure S5, Table S7, Note S7), all of which are reported to be associated with CREBBP.^8^, ^9^ Based on the analysis, we hypothesized that CREBBP may be responsible for the differences in trophoblast development between M16 and T16 embryos.

**FIGURE 4:**
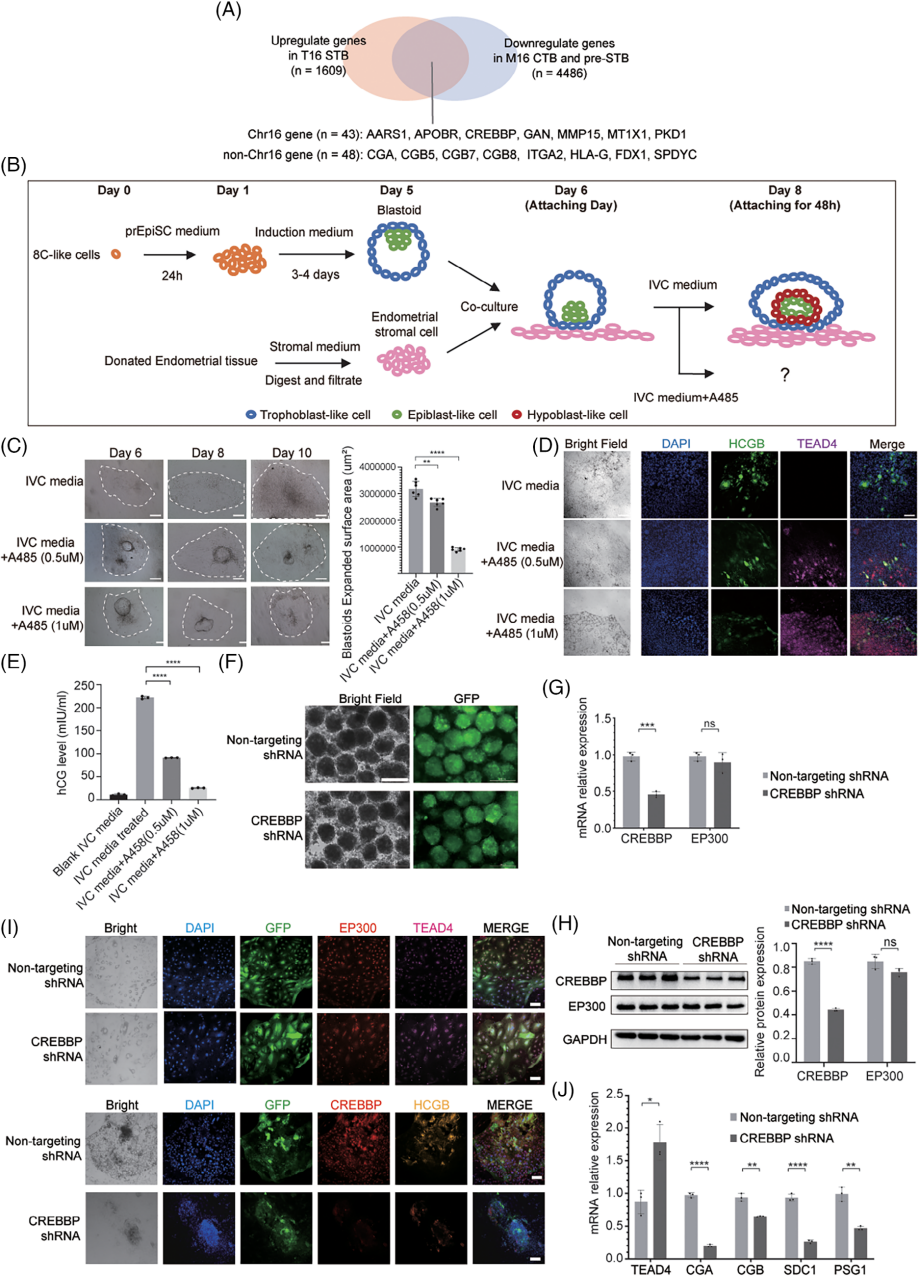
CREBBP supports STB differentiation in peri‐implantation blastoid models. (A) Venn diagram showing the intersection of the DEGs downregulated in M16 CTB and pre‐STB, upregulated in T16 STB; these genes were subsequently filtered for genes located on Chr16 (Table S6). (B) A schematic diagram of blastoid induction. The blastoid model was used to investigate the impact of the CBP/P300 inhibitor A485 on embryonic development and trophoblast differentiation. First, 8C‐like cells were induced to form blastoids, which were then cocultured with endometrial stromal cells. The blastoids adhered to stromal cells and developed into peri‐implantation embryo‐like structures in IVC medium, with or without A485 treatment. The experiment was repeated in three independent biological replicates. (C) The expanded surface area of blastoids decreased after A485 treatment. **p < .005, ****p < 5 × 10e^−5^. (D) Bright field and immunofluorescence microscopy images of cells stained for TEAD4 (TSC marker, purple), hCGB (STB marker, green) and DAPI (nuclei, blue) (scale bars: 50 µm). (E) The hCG level in the blastoid supernatant was tested by a chemiluminescence method. ****p < 5 × 10e^−5^. (F) Lentiviral carrying GFP and CREBBP shRNA were introduced on day 6, and blastoids were cultured until day 10. Robust GFP fluorescence throughout the blastoids confirmed successful viral infection. The experiment was replicated using two distinct CREBBP‐targeting shRNA. The shRNA sequences are provided in Table S8. (G) CREBBP and EP300 mRNA expression after blastoids infected with lentiviral relative to the non‐targeting shRNA condition measured by qPCR. Bars represent mean fold‐change (FC) ± SD. *p < .05, **p < .005, ***p < .0005. (H) Protein levels of CREBBP and EP300 measured by western blot. (I) Representative bright field and immunostaining images of cells stained for TEAD4 (TSC marker), hCG (STB marker), CREBBP and EP300 after blastoids attach on stromal cells (Scale bars: 100 µm). (J) qPCR analysis of TSC marker and STB marker mRNA expression. Bars represent mean fold‐change (FC) ± SD relative to the non‐targeting shRNA condition (*n* = 3 independent experiments).

We used an in vitro blastoid model to elucidate the impact of CREBBP on trophoblast differentiation and implantation (Figure 4B, Figure S6a,b). After A485 (a CREBBP/EP300 inhibitor) was added to the culture system, the expanded surface area of blastoids significantly decreased (Figure 4C), indicating a reduced adhesion capacity of the trophoblast in blastoids. Immunofluorescence revealed a decrease in HCGB expression and an increase in TEAD4 expression following A485 treatment (Figure 4D). Furthermore, there was a significant decrease in the level of hCG secreted in the supernatant of blastoids following A485 treatment (Figure 4E). We also confirmed the above conclusion in TSCs (Figure S6c–e, Note S8). Our results implied that CREBBP/EP300 suppression preserves the CTB state and inhibits differentiation into the STB in TSCs and blastoids, consistent with the trophoblast phenotype observed in M16 embryos.

To rule out the influence of EP300, we specifically investigated the role of CREBBP in trophoblast differentiation. By introducing *CREBBP* shRNA‐carrying lentiviruses into the blastoids, we effectively reduced CREBBP expression without compensation of EP300 (Figure 4F–H). Following CREBBP knockdown, we cultured the blastoids in vitro until day 10. The blastoids exhibited a gene expression pattern similar to M16 with upregulation of TEAD4, and downregulation of hCG, CGA, SDC1 and PSG1, indicating a block in differentiation from CTB to STB (Figure 4I,J). In this unique CREBBP knockdown model with no compensatory upregulation of EP300, we demonstrated that CREBBP plays a crucial role in maintaining STB differentiation.

In conclusion, our study confirmed that aneuploid embryos exhibited diverse developmental abilities at the peri‐implantation stage. We discovered that loss of chr16 can result in abnormal development of the EPI, whereas loss of M22 did not result in this defect. A gain of chr16 caused the premature development of trophoblasts, while a loss of chr16 led to a decrease in trophoblast differentiation. Furthermore, we demonstrated that *CREBBP* is one of the dosage genes affecting STB differentiation at the peri‐implantation stage. CREBBP may have potential applications in assessment of embryo developmental competence, which could help optimize PGT strategies and improve implantation success rates. Our study serves as a reference for peri‐implantation development, offering valuable insights into the molecular characteristics and transitions occurring during early embryo development. This may lay a foundation for further explorations of embryonic defects leading to repeated implantation failure or pregnancy loss.

## AUTHOR CONTRIBUTIONS

Yanwen Xu, Tianqing Li and Chenhui Ding initiated the project. Xueyao Chen performed embryo culture, data collection and wrote the manuscript. Hanwen Yu and Yin Yu performed scRNA‐seq data analysis and wrote the manuscript. Bing Cai performed the blastoids‐related experiments. Gaohui Shi performed the TSC‐related experiments. Yan Xu collected and analysed the PGT data. Lujuan Rong performed embryo staining and photo processing. Boyan Wang performed the blastoids‐related experiments. Canquan Zhou and Jichang Wang provided the guidance and instructions for the project. Chenhui Ding provided clinical samples and technical guidance. Tianqing Li designed and organized the experiments. Yanwen Xu conceived the study and supervised the entire project.

## CONFLICT OF INTEREST STATEMENT

The authors declare no conflicts of interest.

### ETHICS STATEMENT

This study was approved by the Medicine Ethics Committee of The First Affiliated Hospital, Sun Yat‐sen University (LS[2022]No.092). Donated embryos were abnormal blastocysts screened by preimplantation genetic testing for aneuploidy (PGT‐A) or affected embryos determined by preimplantation genetic testing for monogenic disorders (PGT‐M). The informed consent process followed guidelines set by the International Society for Stem Cell Research (ISSCR) and China’s Ministry of Science and Technology and Ministry of Health. The Medicine Ethics Committee of The First Affiliated Hospital, Sun Yat‐sen University, evaluated the scientific merit and ethics of this study. The committee fully reviewed embryo donation and use. All donor couples provided a voluntary informed consent for the research use of surplus embryos in the Department of Reproductive Medicine at The First Affiliated Hospital, Sun Yat‐sen University. No financial compensation was provided. Donor couples were informed that embryos would be used to study human development and donation would not affect their treatment. Culture of all embryos was terminated before d.p.f. 14 to comply with ethical guidelines.

## Supporting information



Supporting Information

Supporting Information

Supporting Information

Supporting Information

Supporting Information

Supporting Information

Supporting Information

Supporting Information

Supporting Information

Supporting Information

## Data Availability

The raw sequence data of single‐cell RNA‐sequencing in this study have been deposited in the Genome Sequence Archive^10^ in National Genomics Data Center^11^, China National Center for Bioinformation / Beijing Institute of Genomics, Chinese Academy of Sciences (GSA‐Human: HRA005378) that are publicly accessible at https://ngdc.cncb.ac.cn/gsa‐human. The scRNA‐seq data of human peri‐implantation embryos (for Figure 3D, Figure S3d, ref^3^) is downloaded from GEO: GSE136447. The custom codes used for the data analyses is now available in our GitHub repository (https://github.com/AIBio/CXY_Aneuploid_scRNA‐seq).
