# Supplementary material for: Abnormal lineage differentiation of peri‐implantation aneuploid embryos revealed by single‐cell RNA sequencing
Source: Clin Transl Med. 2025 May 7;15(5):e70326. doi: 10.1002/ctm2.70326 (PMC12056499; doi:10.1002/ctm2.70326)
Supplement: Supplementary file 1 — Supporting Information [file CTM2-15-e70326-s009.docx]

**Letter to the Journal (**Previous submission number: CTM2-2025-02-0581**)**

**Abnormal lineage differentiation of peri-implantation aneuploid embryos revealed by single-cell RNA sequencing**

Xueyao Chen^1,4,5,#^, Hanwen Yu^3,6,7,#^, Yu Yin^2,#^, Bing Cai^1,4,5,#^, Gaohui shi^2,#^, Yan Xu^1,4,5^, Lujuan Rong^2^, Xiu Yu^3,6,7^, Boyan Wang^1,4,5^, Canquan Zhou^1,4,5^, Jichang Wang^1,3,6,7^, Chenhui Ding^1,4,5,*^, Tianqing Li^2,*^ & Yanwen Xu^1,4,5,*^

^1^ Center for Reproductive Medicine, Department of Gynecology & Obstetrics, the First Affiliated Hospital, Sun Yat-Sen University, Guangzhou, Guangdong, China

^2^ Yunnan Key Laboratory of Primate Biomedical Research, Institute of Primate Translational Medicine, Kunming University of Science and Technology, Kunming, Yunnan, China

^3^ Advanced Medical Technology Center, The First Affiliated Hospital, Zhongshan School of Medicine, Sun Yat-sen University, Guangzhou, Guangdong, China

^4^ Guangdong Provincial Key Laboratory of Reproductive Medicine, Guangzhou, Guangdong, China

^5^ Guangdong Provincial Clinical Research Center for obstetrical and gynecological diseases, Guangzhou, Guangdong, China

^6^ Key Laboratory for Stem Cells and Tissue Engineering (Sun Yat-sen University), Ministry of Education, Guangzhou, Guangdong, China

^7^ Department of histology and embryology, Zhongshan School of Medicine, Sun Yat-sen University, Guangzhou, Guangdong, China

^#^ These authors contributed equally: Xueyao Chen, Hanwen Yu, Yu Yin, Bing Cai, Gaohui shi

^*^ Email: dchenh@mail.sysu.edu.cn; litq@lpbr.cn; [xuyanwen@mail.sysu.edu.cn](mailto:xuyanwen@mail.sysu.edu.cn)

**Key words**: Embryo development; aneuploidy; trophoblast differentiation; single cell transcriptome; blastoid

## Materials and methods

### Ethics statement

This study was approved by the Medicine Ethics Committee of The First Affiliated Hospital, Sun Yat-sen University (LS[2022]No.092). Donated embryos were abnormal blastocysts screened by preimplantation genetic testing for aneuploidy (PGT-A) or affected embryos determined by preimplantation genetic testing for monogenic disorders (PGT-M). The informed consent process followed guidelines set by the International Society for Stem Cell Research (ISSCR) and China’s Ministry of Science and Technology and Ministry of Health. The Medicine Ethics Committee of The First Affiliated Hospital, Sun Yat-sen University, evaluated the scientific merit and ethics of this study. The committee fully reviewed embryo donation and use. All donor couples provided a voluntary informed consent for the research use of surplus embryos in the Department of Reproductive Medicine at The First Affiliated Hospital, Sun Yat-sen University. No financial compensation was provided. Donor couples were informed that embryos would be used to study human development and donation would not affect their treatment. Culture of all embryos was terminated before d.p.f. 14 to comply with ethical guidelines.

### Inclusion criteria and embryo thawing

Blastocyst screened as aneuploidy (T16, M16, T21, M21, T22 and M22) or affected with a monogenic disease were included in the study. According to the Gardner’s scoring system, blastocysts with an expansion degree and hatching status between grade 3 and 5, and a visible inner cell mass above grade B, were thawed. Human embryo culture medium G-2 (10029, Vitrolife) was equilibrated overnight before embryo thawing. Several drops of 30 µl G-2 medium were added to a 3.5 cm dish (LG18-101C-1, Corning) and covered with 2 ml of mineral oil (10029, Vitrolife). G-2 medium was placed in a 37°C incubator overnight. Human blastocysts (d.p.f 5-6) were thawed using a home-made thawing solution. After culturing in drops of the equilibrated G-2 medium for 4 hours, embryos were transferred to acidic Tyrode’s solution (T1788, Sigma-Aldrich) to remove the zona pellucida. Once the zona pellucida vanished, embryos were immediately transferred back to G-2 medium. Embryos were washed in G-2 medium twice and then transferred to the in vitro culture medium.

### In vitro three-dimensional culture of human embryos

The *in vitro* culture protocol of human embryos was followed the method of Xiang *et al*^1^. In detail, embryos without a zona pellucida were cultured in a low attachment 96-well plate (3474, Corning) with one embryo and 150μL G-2 medium in each well. Embryos were cultured at 37°C, 6% CO_2_, and saturated humidity. Initially, from d.p.f 6 to 8, culture medium was replaced with modified *in vitro* culture medium 1 (mIVC1). On d.p.f 8, 50% of the mIVC1 medium was replaced with modified *in vitro* culture medium 2 (mIVC2). On d.p.f 9, embryos were transferred to new wells containing mIVC2 plus 10% Matrigel (354277, Corning). Thereafter, 50% of the culture medium was replaced daily with fresh mIVC2 plus 10% Matrigel. mIVC1 and mIVC2 were pre-equilibrated in the incubator for at least 4 hours before use.

The composition of mIVC1 included: advanced DMEM/F12 (12634-010, Thermo Fischer Scientific) supplemented with 20% (vol/vol) DFBS (defined fetal bovine serum)(bs-0003, Biosera), 2 mM L-glutamine (25030, Thermo Fisher Scientific), 1x ITS-X (51500-056, Thermo Fisher Scientific), 8 nM β-estradiol (E8875, Sigma-Aldrich), 200 ng/ml progesterone (P0130, Sigma-Aldrich), 25 μM N-acetyl-L-cysteine (A7250, Sigma-Aldrich), 0.22% (vol/vol) sodium lactate (L7900,Sigma-Aldrich), 1 mM sodium pyruvate (P4562, Sigma-Aldrich) and 10μM Y27632 (S1049, Selleck). The composition of mIVC2: advanced DMEM/F12 supplemented with 30% (vol/vol) KOSR (knockout serum replacement) (A3181501, Thermo Fischer Scientific), 2 mM L-glutamine, 1x ITS-X, 8 nM β-estradiol, 200 ng/ml progesterone, 25 μM N-acetyl-L-cysteine, 0.22% (vol/vol) sodium lactate, 1 mM sodium pyruvate and 10μM Y27632.

### Frozen section immunostaining

Embryos were fixed by 4% paraformaldehyde (PFA), washed in PBS, and dehydrated by 15% sucrose for 5 min. They were then embedded in optimal cutting temperature (OCT) compound. Embedded embryos were sectioned into 10 μm slices using a Leica frozen slicer. Before immunostaining, slides were washed in PBS to remove OCT and permeabilized by 0.2% Triton X-100 for 30 minutes at room temperature. After blocking with 3% bovine serum albumin (BSA) in PBS for 2 hours at room temperature, sections were incubated with primary antibodies overnight at 4°C and then washed three times with 0.05% Tween-20. The sources, catalog number, and dilution of primary antibodies were: mouse anti-OCT3/4 (Santa Cruz, SC5279, 1:400), rabbit anti-Cytokeratin 7 (CK7) (Abcam, AB181598, 1:300), Mouse anti-hCG (Abcam, AB9582, 1:100), goat antiNANOG (Abcam, AB80892, 1:250), mouse anti-HLA-G (Abcam, AB52455, 1:200), goat anti-GATA6 (R&D Systems, AF1700, 1:200), Phalloidin (F-actin) Alexa Fluor®488 (Thermo Fisher Scientific, A12379, 1:1000). Secondary antibodies were incubated at room temperature for 2 hours. Slices were washed three times with 0.05% Tween-20. Images were captured using Leica SP8 laser confocal microscope.

### Whole embryo staining and 3D reconstruction

Fixed embryos were permeabilized using 0.5% Triton X-100 in PBS, incubated overnight at 4°C. They were then blocked with 3% BSA in PBS for 4 hours at room temperature and transferred to new wells. After incubating with primary antibodies for 16-18 hours at 4°C, embryos were washed three times in PBS containing 0.05% Tween-20. They were then incubated with secondary antibodies for 4 hours at room temperature. Finally, embryos were washed three times in PBS with 0.05% Tween-20 and then transferred to wells of 8-well IbiTreat μ-plates (IB-80826, Ibidi) containing 60% glycerol aqueous solution for imaging.

### Induction of blastoids

Human preimplantation epiblast-like stem cell（prEpiSC）were obtained from Jichang Wang’s lab. The induction process is according to Jichang Wang lab’s protocal^2^. PrEpiSC were then cultured in the modified G-2 PLUS medium containing 10 ng/mL human LIF, 5 ng/mL BMP4, 1.5 mM PD0325901, 0.5 mM LPA, and 1 mM A83-01 for 6 days to form the blastoids.

### Endometrial stromal cell culture and plating

The endometrial tissue was sourced from voluntary donations by patients undergoing endometrial biopsies. Biopsies were washed, minced, and enzymatically digested with 0.4mg/mL Collagenase V (Sigma) and 1.25 U/mL Dispase II (Sigma). Digestion suspension was neutralized and passed through a 100 mmcell strainer to separate large glandular tissue fragments and then a 40 mm cell strainer. The filtrate from glandular fragment separation was further passed through a 10 mm cell strainer to separate stromal cells. Filtrate was centrifuged and resuspended in stromal culture medium containing 10% fetal bovine serum (FBS, Gibco) in DMEM/F12 and transferred to a T25 tissue flask and incubated for 15 min. The medium suspension was then moved to a new T25 tissue flask to separate out contaminating epithelial cells. These proliferate ESC cells then be passaged onto glass coverslips using for co-culture with blastoids.

### Blastoid extended culture and cocultured with endometrial stromal cell

The glass coverslips coated with ESC cells were placed in a petri dish. The day 6 blastoids were transferred onto the petri dish and cocultured with ESC cells. From day 6 to 8, culture medium was the modified *in vitro* culture medium 1 (mIVC1). On day 8, 50% of the mIVC1 medium was replaced with modified *in vitro* culture medium 2 (mIVC2). On day 9, the medium was replaced by mIVC2. Thereafter, 50% of the culture medium was replaced daily with fresh mIVC2. Blastoids were assessed on day 8-10. mIVC1 and mIVC2 were pre-equilibrated in the incubator for at least 4 hours before use. The composition of mIVC1 and mIVC2 was described in the section “In vitro three-dimensional culture of human embryos”.

### Human Chorionic Gonadotropin Quantification

2ml of each group spent medium was collected and snap frozen in liquid nitrogen and stored at -80℃. 500ul of Samples were analyzed for hCG using electrochemiluminescence on a RocheCobase601 analyzer.

### Outgrowth Area Measurement

Images of peri-implantation blastoids were captured using an BioTek Cytation 5 (Agilent Technologies) camera fixed to a stereomicroscope. The measurement software provided with the machine outputted the corresponding migration area using the scale bar as a reference.

### Blastoids Immunofluorescence Staining

Blastoids were grown on ESC glass coverslips (Nest biotechnology, cat#801008). The adherent cells and blastoids were all fixed with fresh 4% paraformaldehyde/phosphate buffer for 10 minutes at room temperature, washed three times with DPBS, and permeabilized in 0.2% Triton/DPBS for 30 minutes. Cells were blocked with 5% BSA/DPBS and incubated with primary antibodies diluted in 1% BSA/DPBS overnight at 4^。^C. Cells were then washed three times with DPBS, incubated with secondary antibodies for one hour at room temperature, washed with DPBS, mounted by Fluoroshield with DAPI (Sigma, catalog. no. F6057) and imaged with a confocal microscope (Zeiss 880). The following antibodies were used at the indicated dilutions: rabbit anti-TEAD (1:200), mouse anti-HCG (1:200), goat anti-gata3 (1:200), anti-gata6 (1:200), anti-CK19 (1:200). Samples were washed three times with wash buffer under gentle orbital shaking for 15 minute ,followed by incubation with fluorescently conjugated secondary antibodies diluted (1:400) in blocking buffer for 1h at 37^。^C. Samples were washed three times with wash buffer as described above. Finally, cells were counterstained with 300 nM 40,6-diamidino-2-phenylindole (DAPI) solution at room temperature for 10 mins.

### TSCs Differentiation

A 6-well plate was coated with 5 mg/ml Col IV at 37°C for at least one hour. TSCs were seeded in the 6-well plate at a density of 0.5-1×10^6^ cells per well and cultured in 2 mL of TS medium [DMEM/F12 supplemented with 0.1 mM 2-mercaptoethanol, 0.2% FBS, 0.5% Penicillin-Streptomycin, 0.3% BSA, 1% ITS-X supplement, 1.5 μg/ml L-ascorbic acid, 50 ng/ml EGF, 2 μM CHIR99021, 0.5 mM A83-01, 1 μM SB431542, 0.8 mM VPA and 5 μM Y27632]. Cells were cultured at 37°C 5% CO_2_ and the culture medium was replaced every two days. When cells reached 60%–80% confluence, they were dissociated with TrypLE for 10-15 min at 37°C and passaged to a new Col IV-coated 6-well plate at a 1:2-1:4 split ratio.

For the induction of STB cells, TSCs were seeded in a 6-well plate pre-coated with 2.5 mg/ml Col IV at a density of 1-3×10^5^ cells per well, and cultured in 2 mL of ST medium [DMEM/F12 supplemented with 0.1 mM 2-mercaptoethanol, 0.5% Penicillin-Streptomycin, 0.3% BSA, 1% ITS-X supplement, 2.5 μM Y27632, 2 μM forskolin, and 4% KSR]. The medium was replaced at day 3, and the cells were analyzed at day 5.

### Short hairpin RNA (shRNA) transduction.

High-titer HIV-1 based VSV G-pseudotyped vector stocks with *CREBBP* or nontargeting shRNA sequences were produced by transient transfection of HEK293T cells. Viral infection was performed at a multiplicity of infection of 10 for 24 hours. Two days post-infection, the cells showed strong GFP fluorescence, confirming efficient infection.

### RNA extraction, cDNA synthesis and qPCR

RNA was extracted using the RNeasy mini kit (Qiagen, 74106) and cDNA synthesis was performed using the Superscript III (Invitrogen, 18080093) enzyme. Quantitative PCR (qPCR) was performed using the ChamQ Universal SYBR qPCR Master Mix (Vazyme, Cat#Q711) on the LightCycle (Roche, LightCycle96). Quantification was performed using Microsoft Office Excel by applying the comparative Cycle threshold (Ct) method. Relative gene expression levels were normalized to GAPDH.

### Isolation of single cells

Embryos were washed in DPBS three times, followed by two washes in TrypLE (12605010, Gibco). After incubation in TrypLE for 15 min at 37 °C, embryos were transferred to DPBS, and dissociated into single cells by repeated pipetting. Remained cell clumps were incubated in TrypLE for an additional 10 minutes. Isolated single cells were washed in DPBS three times and picked up into lysis buffer. All above operations were performed under a Nikon SMZ645 microscopy.

### RNA-sequencing of single cells

Synthesis and amplification of full-length cDNAs were performed following Smart-seq2 protocol^3^. Reverse transcription reactions and cDNA amplifications were performed using SuperScript II (18064071, Invitrogen) and KAPA HiFi HotStart ReadyMix (KK2601, KAPA Biosystems), respectively. Amplification products quality was assessed by Bioanalyzer 2100. We selected samples as target products present in the 1~2kbp fragment length, with no or few multi-peak fragments, and no obvious small fragments.

The cDNA was amplified for PCR analysis. Given the limited number of EPI (NANOG-positive) and hypoblast cells (PDGFRA-positive), we selected all PCR-identified cells from these two populations, along with a randomly chosen subset of trophoblast cells (GATA3-positive) for scRNA-seq. For PCR double marker-positive cells, we selected all the cells that were either NANOG or PDGFRA positive, or both markers positive. This approach aimed to enrich the epiblast and hypoblast populations, which are limited in number at this developmental stage.

Library construction and sequencing were then performed on qualified single cell samples by Annoroad Gene Technology (http://www.annoroad.com/). Sequencing was performed on the Illumina NovaSeq 6000.

### Quality control and processing of scRNA-seq data

Quality of all high-throughput sequencing data were first comprehensively checked by package FastQC (release 0.12.1; default parameter) whose results were parsed with packge MultiQC (release 1.17; default parameter)^4^. Next, adapter sequences and bases with sequencing quality ≤ 25 were trimmed from raw data by TrimGalore (release 0.6.5; default parameter) and then the trimmed sequences shorter than 30 bp were filtered. Trimmed read pairs were aligned to genome (hg38) with STAR (release 2.7.4a)^5^ . For quantification of gene and repeat locus expression, the following command-line arguments were used: --outSAMmapqUnique 255. We computed the number of mapping reads unambiguously attributed to each gene and repeat locus using the featureCounts package (release 2.0.6; default parameter)^6^.

### Clustering and identification of cell types

We utilized the Seurat toolkit (release 3.2.0)^7^ to perform downstream analysis of scRNA-seq data. The overall quality of our data was good, with only one cell failing to meet the percent.mt < 20% criterion and just four cells not adhering to the 4000 < nFeature_RNA < 15000 range, while the majority satisfied these conditions. Our dataset includes embryos with chromosomal anomalies, which could lead to reduced cell quality due to developmental arrest. This may result in increased mitochondrial content and fewer gene count. To maintain the integrity of this variability, we opted to retain all 717 cells without exclusion in this procedure.

Then, we normalized and scaled the count data using “NormalizeData” (scale factor = 10,000) and “ScaleData” function with default parameters. We performed principle component analysis (PCA) on the top 2000 highly variable genes (HVGs) using ‘‘RunPCA’’ function. Then, we selected the significant principle components according to the p-values produced by “ScoreJackStraw” and standard deviation for downstream analysis. Cells were then clustered utilizing the ‘‘FindClusters’’ function by embedding cells into a graph structure in PCA space. The clustered cells were then projected onto a two-dimensional space using “RunUMAP” function. To annotate cell clusters, we first identified the differentially expressed genes on each cluster with “FindMarkers” function. These cell clusters were then manually annotated according to curated known cell markers. The cell clusters consistently expressing the same cell marker were merged. Lastly, we utilized the function “CellCycleScoring” to define the cell cycle and then visualized the results with bar plots.

### Differential gene expression and enrichment analysis

Differential expression analyses of scRNA-seq across different groups were conducted by edgeR (release 3.40.2)^8^ ran in the R studio interface (R release 4.2.3). Differentially expressed genes were selected with minimum threshold of 0.58 in absolute log2 fold change and *P* value < 0.05. Differentially expressed genes were then filtered for the minimum threshold of 0.005 in the average normalized count. Functional enrichment analyses were performed by R package clusterProfiler (release 4.6.2)^9^.

### Integrated, correlation and trajectory analysis

Integrating single-cell data across experiments, including data generated in this study and accessed from public dataset (GSE136447), was performed by R package Harmony (release 1.1.0)^10^. The cosine correlation index was calculated by R package CoreGx (release 2.2.0)^11^ and then visualized by R package ComplexHeatmap (release 2.14.0)^12^. Finally, the integrated data was used as input of trajectory analysis with R package monocle3 (release 1.3.4)^13^.

### Regulatory analysis of scRNA-seq data

To investigate the activities of TFs, we performed SCENIC (release 0.12.1)^14^ analysis on scRNA-seq data of human embryos in different developmental stage and cell types. Firstly, we apply a regression per-target approach to identify the co-expression modules by analyzing the input single-cell expression matrix of gene. Then the indirect targets were filtered based on cis-regulatory motif scanning and ranking results. The activities of these TFs were quantified with AUCell algorithm by enriching and scoring the target genes of the regulators, which obtains the regulon activity score. Finally, we visualized the gene regulatory networks by Cytoscape package (release 3.9.1)^15^.

### Copy number variation analysis

InferCNV (release 3.14) was used to estimate CNV of all cells. Euploid embryo cells from a preimplantation genetic testing for aneuploidy (PGT-A)-validated euploid embryo were used as reference cells. Denoising by Hidden Markov Model (HMM) step was performed with a cutoff of 1 for gene selection in ‘subclusters’ analysis mode. CNV scores of genes in chromosome 16 were selected and visualized using the VlnPlot function of Seurat in R.

### RNA velocity analysis

Velocyto (release 0.17.17) were used to calculate RNA velocity from single-cell RNA sequencing data and convert sorted BAM file into a loom file. The velocyto.R(v0.6) package exported new count matrices from the loom file to R. RunVelocity from the SeuratWrappers package calculated RNA velocity with default parameters. show.velocity.on.embedding.cor in Velocyto.R visualized transcriptome dynamics with adjusted arrow lengths.

### Statistical analysis

Variability between experimental replicates was estimated using standard error, calculated from a minimum of three independent embryos unless otherwise noted. Statistical significance was determined for changes observed between groups. Differences in cell number were evaluated using an unpaired two-sample Student’s *t*-test, relying on Graphpad software and a *P*-value of less than 0.05. As gene expression data did not follow a normal distribution, Wilcoxon rank sum test was applied to analyze differences between cell populations characterized by distinct patterns of pathway activation.

## Supplementary Note

### Supplementary Note 1

The post-implantation development of euploid embryos exhibited normal morphology. The epiblast enveloped to form the amniotic cavity. The epiblast and hypoblast together contributed to the formation of the bilaminar germ disc (Fig. 1b). The large and fused syncytiotrophoblast (STB) also became distinctly apparent. The structure of embryos cultured *in vitro* up to d.p.f. 12 was consistent with the *in vivo* morphological structure of early embryos at Carnegie stage 5^18^, confirming the accurate modeling of normal developmental processes *in vivo* at the peri-implantation stage.

### Supplementary Note 2

The embryo karyotype was determined by PGT at d.p.f. 5 or 6. It is accepted that trophectoderm biopsy could largely represent the karyotype of the entire embryo. To verify the embryo karyotype, we used the R package inferCNV to predict the karyotype based on scRNA-seq. The CNV prediction karyotypes were in 94.44% agreement with the PGT results (n=17/18). The results for only one embryo (No. M16#3) were inconsistent: PGT revealed M16 at d.p.f. 6 (Supplementary Fig. 2a, b), while scRNA-seq indicated euploidy at d.p.f. 8 (Fig. 2a, red arrow). The S.D. value of this embryo’s NGS from PGT diagnosis was 7.469, indicating low-quality whole-genome amplification in PGT. Among the 59 cells analyzed by scRNA-seq in this embryo (No. M16#3), the majority exhibited a CNV score consistent with euploidy, except for one trophoblast cell, which had half the CNV score, showing that it remained M16 (Supplementary Fig. 2c, red arrow). The transcriptomic profiles of euploid cells in this mosaic embryo were similar to those of other euploid embryos, while the transcriptomes of M16 cells more closely resembled those of other M16 embryos (Supplementary Fig. 2d, red arrow). Due to the uncertainty regarding the karyotype of this embryo (No. M16#3), we use "M16?" to refer to its karyotype in the subsequent text. The embryo was excluded from subsequent differential expression analysis.

### Supplementary Note 3

Using GSEA, we aggregated dysregulated pathways across karyotypes and presented those pathways which were significantly altered (*P* < 0.05) in two or more karyotype comparisons (Fig. 2g). It revealed the consistent downregulation of the pentose phosphate pathway, alanine, aspartate, and glutathione metabolism pathway across the M16, M22, and T16 embryos. Metabolism-related pathways such as oxidative phosphorylation, amino acid biosynthesis, carbon metabolism, purine metabolism, and cofactor biosynthesis, along with cell cycle-related pathways such as mismatch repair, base excision repair, and DNA replication, were exclusively upregulated in T16 embryos but downregulated in M16 and M22 embryos. The cell adhesion molecule pathway was upregulated in M22 embryos. Ovarian steroidogenesis was upregulated in both M22 and T16 but downregulated in M16. Pathways that were upregulated across all three karyotypes included the FoxO signaling pathway and the phosphatidylinositol signaling system pathway.

### Supplementary Note 4

To investigate the distribution of DEGs across chromosomes, we quantified the DEG ratio (DEG number / chromosome total gene number) and classified the DEGs according to their fold changes. For the T16 and M16 embryos, the highest DEG ratio was found on Chr16 (Fig. 2h). For M22 embryos, the chromosome with the greatest DEG ratio was Chr22. The fold changes in the expression of these genes ranged from 1.5 to 3, which indicated that chromosomal dosage predominantly affected gene expression.

### Supplementary Note 5

We performed differential expression analysis within specific cell types (Supplementary Fig. 4a). Overall, downregulated DEGs outnumbered upregulated ones, with T16 D10 STB exhibiting the highest number of upregulated DEGs and M16 pre-STB the most downregulated. M16 and M22 embryos shared greater DEG overlap than other groups (Supplementary Fig. 4b). Pathway enrichment analysis revealed key dysregulated pathways relevant to trophoblast development (Supplementary Fig. 4c, Supplementary Table 5). Wnt signaling, crucial for trophoblast lineage specification, was downregulated in T16 but upregulated in M16 CTB and pre-STB. This suggests that T16 may suffer from impaired Wnt-driven differentiation, whereas M16 trophoblast cells may remain in a progenitor-like state. FoxO signaling, a regulator of oxidative stress and trophoblast survival, was broadly disrupted, implying defects in cellular stress adaptation. T16 STB exhibited significant downregulation of cell adhesion, focal adhesion, and ECM-receptor interaction pathways, which are essential for implantation. These changes likely indicate impaired maternal-fetal interface establishment. M16 CTB and pre-STB upregulated Hippo signaling and pathways regulating stem cell pluripotency. Hippo signaling is known to maintain trophoblast progenitors, and its upregulation may contribute to the failure of M16 cells to transition toward differentiated lineages. Conversely, T16 EPI showed marked downregulation of Hippo signaling pathway, suggesting a loss of epiblast plasticity, potentially leading to developmental stagnation. Metabolic disruptions were evident particularly in M16 embryos.

### Supplementary Note 6

Integrating our scRNA-seq data with the data from the published dataset^1^ , the correlation analysis demonstrated that the CTB of M16 was more closely aligned with the early TE, while the pre-STB of M16 more closely resembled the CTB defined in Xiang's dataset (Fig. 3f brown frame). The STB of T16 was close to the mature STB at later stages (Fig. 3f, chartreuse frame). In addition, aneuploid EPIs and hypoblasts exhibited no significant advances or delays in differentiation. Pseudotime analysis also suggested that the M16 trophoblast was at an earlier stage of differentiation, while the T16 trophoblast was at a more advanced stage of differentiation (Supplementary Fig. 3d).

### Supplementary Note 7

Based on TFs activity analysis through SCENIC, we found that the transcriptional factors CEBPA and ELF3 were among the top 10 RSS transcription factors for both the T16 and M16 groups (Supplementary Fig. 5a). FOXO1 and CEBPB exhibited significant differences in TF activity between aneuploid and euploid embryos (Supplementary Fig. 5b, Supplementary Table 7). The target genes of ELF3 and FOXO1 were enriched in pathways related to placental development (Supplementary Fig. 5c). The expression of the target genes of CEBPB (CGB5), ELF3 (CITED2 and OVOL1 related to trophoblast development^19–21^), and FOXO1 (SOX17 and GATA4 related to lineage differentiation), displayed contrasting patterns in T16 STB and M16 CTB compared to euploid controls (Supplementary Fig. 5d), which suggests that they may be influenced by the secondary effects of dose-dependent genes. CREBBP interacts with C/EBPα and C/EBPβ (encoded by CEBPA and CEBPB, respectively)^22–27^. In addition, CREBBP exhibited acetylation activity that upregulated ELF3 expression^28^ while suppressing FOXO1 activity^29,30^. Thus, we propose that CREBBP regulates downstream STB differentiation by modulating these TFs.

### Supplementary Note 8

CREBBP/EP300 inhibitors A485 or CPI637 were added to STB medium during the process of inducing the transformation of TSCs into STBs. In accordance with previous study^31^, we observed that differentiation of STBs was significantly inhibited after adding CREBBP/EP300 inhibitors. Under the bright field, compared to those in the STB-induced differentiation group, cells treated with the inhibitors exhibited more epithelial-like morphology and did not aggregate to form syncytia, which were similar to TSCs (Supplementary Fig. 6c). Immunofluorescence staining revealed a significant decrease in HCGB expression, while CDH1 and TEAD4 increased to levels nearly comparable to those in TSCs (Supplementary Fig. 6d). qPCR revealed that CTB markers (*TP63*, *NR2F2*, and *TEAD4*) were significantly downregulated in the process from TSC to STB but significantly rebounded after the addition of inhibitors. In contrast, the expression of STB markers (*CGA*, *CGB*, *PSG1*, and *SDC1*) increased significantly from the TSC to the STB stages but decreased significantly after the addition of the inhibitors (Supplementary Fig. 6e). Our results demonstrated that inhibition of CREBBP/EP300 can significantly inhibit the differentiation of STBs.

### Supplementary Note 9

To further explore the role of CREBBP in STB differentiation, we knocked down *CREBBP* in TSCs by lentiviral infection (Supplementary Fig. 6f). Similar to the previous study, we failed to observe a significant impact on TSCs after CREBBP knockdown. Notably, we detected an increase in EP300 following CREBBP knockdown, indicative of a potential compensatory mechanism that mitigates the impact of CREBBP decrease (Supplementary Fig. 6g). KAT8, another histone acetyltransferase implicated in trophoblast differentiation^73^, also showed an increase after CREBBP knockdown (Supplementary Fig. 6g). Therefore, we speculated that the impact of CREBBP knockdown on TSCs may be compensated by upregulation of EP300 and KAT8, thus preserving the differentiation potential of TSCs.

### Supplementary Note 10

Our study has several limitations: 1. The *in-vitro* embryo culture system exhibits inherent differences from *in-vivo* development and cannot fully recapitulate physiological embryogenesis. 2. Blastoids and TSCs display molecular and functional disparities compared to real embryos, limiting their fidelity in modeling developmental processes. 3. Due to the low-throughput nature of Smart-seq2, we captured only a limited number of epiblast cells, precluding robust characterization of aneuploid epiblast transcriptomes. 4. Given the high homology between CREBBP and EP300, compensatory effects of EP300 upon CREBBP perturbation cannot be excluded. We anticipate that future studies will overcome these limitations and enable more comprehensive investigations in this field.

## Supplementary Figures


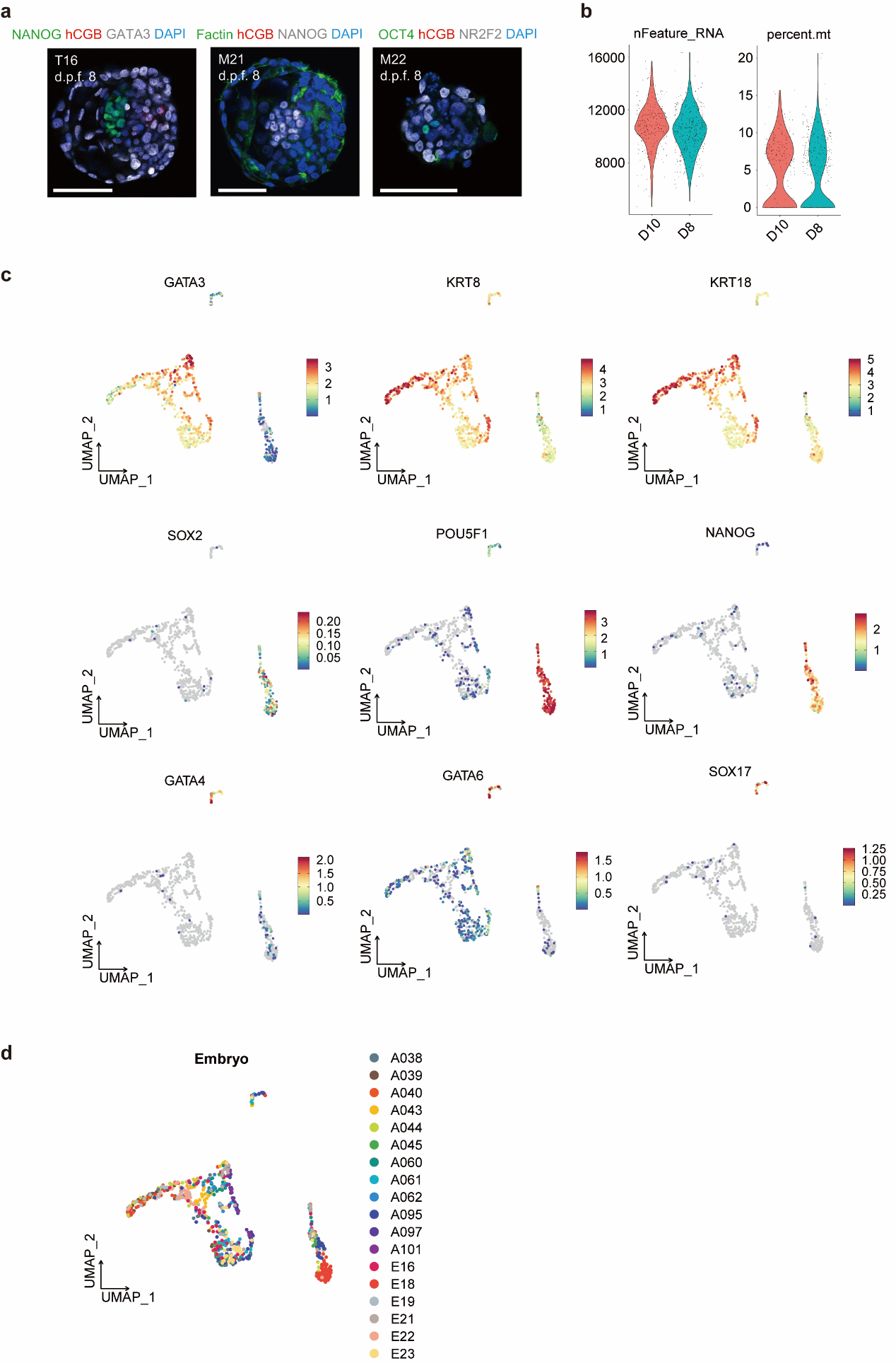


**Supplementary Figure 1**. The morphology of aneuploid embryos, the quality control of single cell RNA sequencing and the feature plots of lineage markers. **(a)** The immunostaining of aneuploid embryos at d.p.f. 8. Few hCGB (+) STB cells were observed in the monosomy embryos, indicating that cell differentiation into STBs was restricted. Scale bar: 100μm. **(b)** Quality control of single cell RNA sequencing. **(c)** The feature plot illustrates the expression patterns of lineage-specific genes. (d) The UMAP plot distinguishing individual embryos.


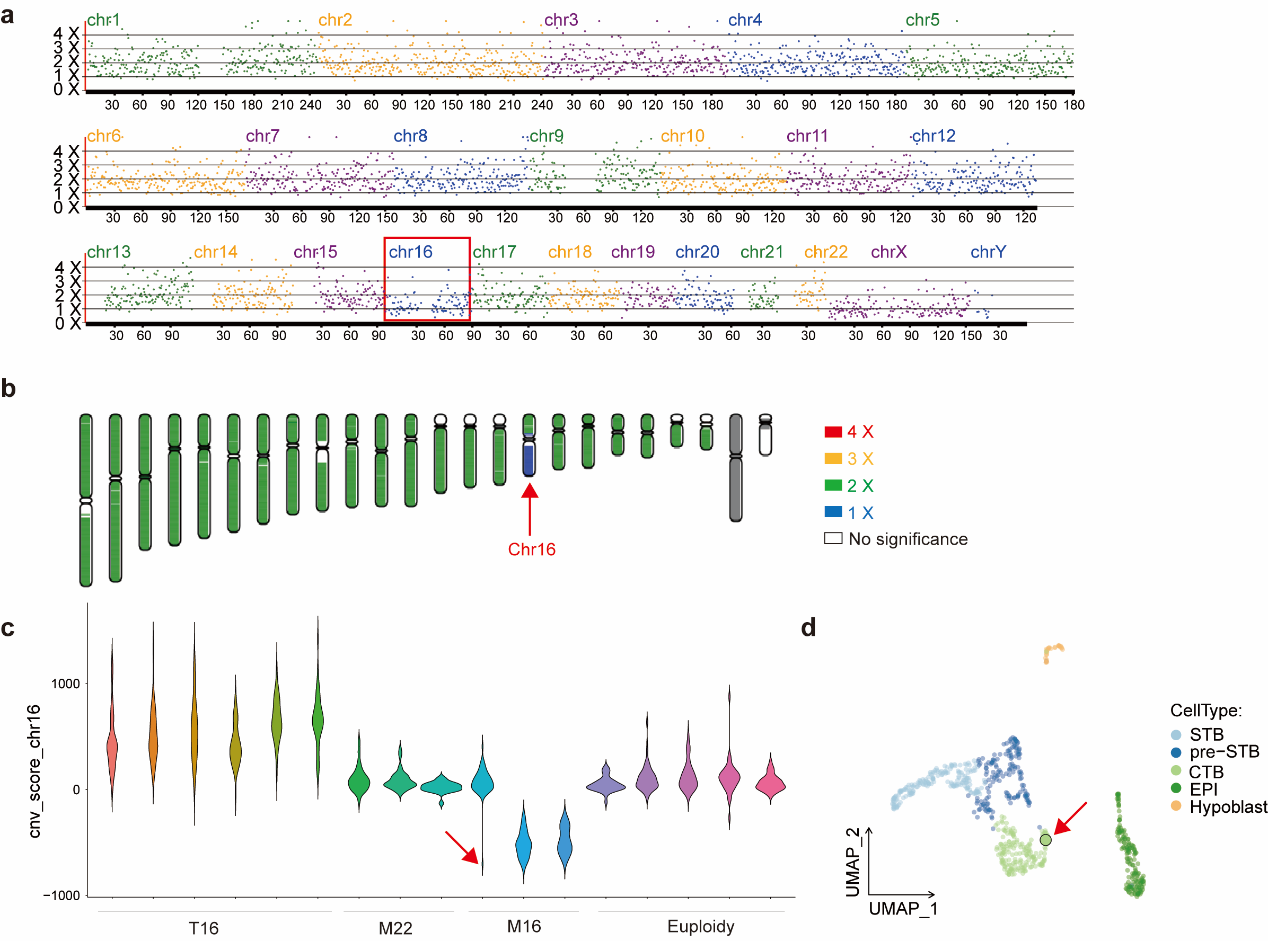


**Supplementary Figure 2**. Verification of the embryonic karyotype based on scRNA-seq. **(a,b)** The chromosome dot plot and the diagnosis chromosome schematic diagram by PGT-A of the mosaic embryo (No. M16#3). The number of gene copies on chromosome 16 decreased by half. **(c)** Violin plot of the single-cell CNV score on chr 16 of embryos. According to the CNV score, we inferred that most cells in embryo No. M16#3 were euploid, with only one cell remaining, M16 (red arrow). **(d)** The only M16 cell in the mosaic embryo (No. M16#3) was a trophoblast cell (red arrow).


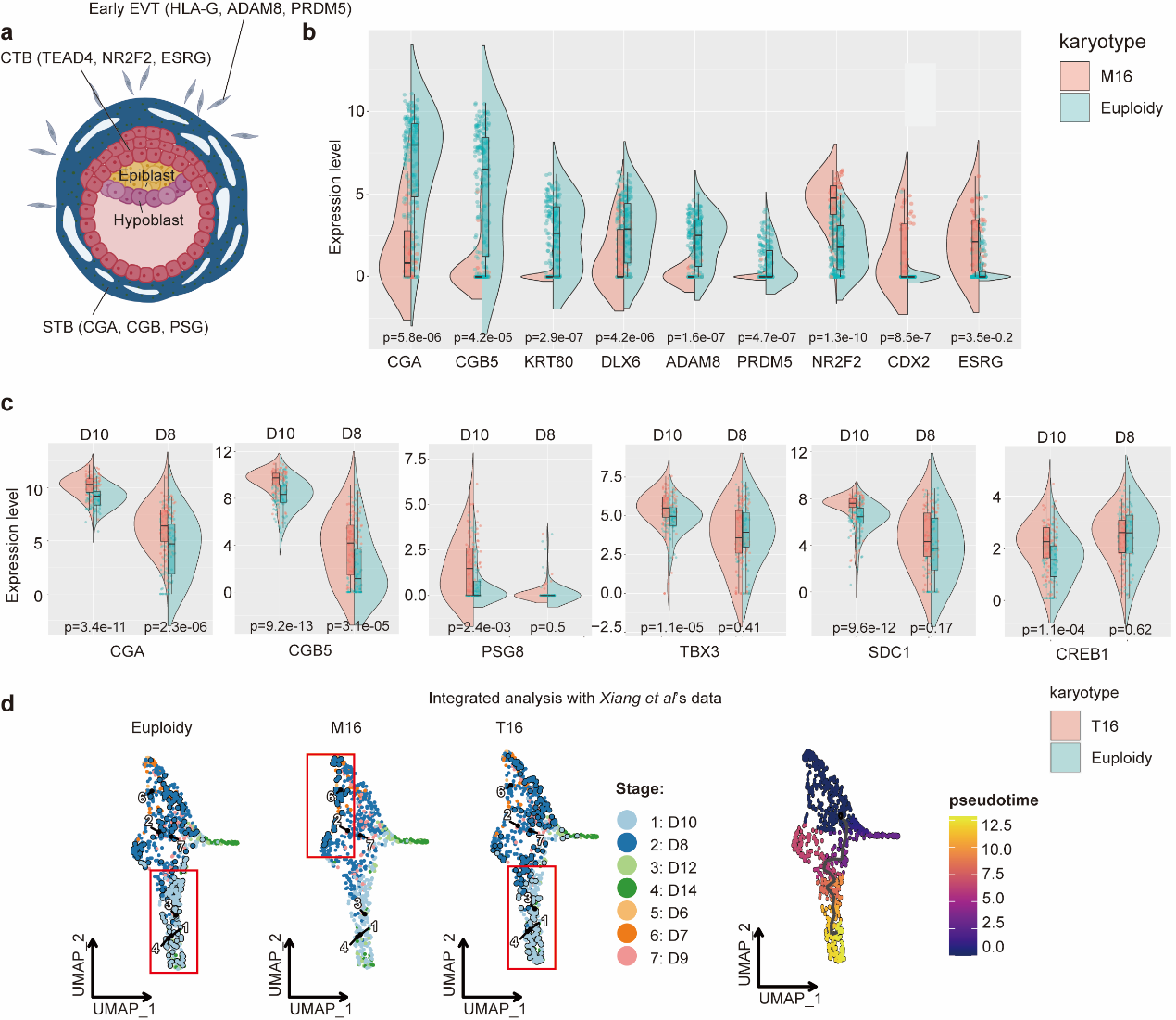


**Supplementary Figure 3**. The abnormal differentiation of trophoblast in aneuploid embryos. (a) Schematic representation of trophoblast differentiation during peri-implantation period. Cytotrophoblasts (CTBs, expressing TEAD4, NR2F2, and ESRG) represent the proliferative trophoblast population. Polar CTBs undergo proliferation and subsequent cell fusion to form syncytiotrophoblasts (STBs, expressing CGA, CGB and PSG), which progressively expand to envelop the developing embryo. At the embryonic pole facing the endometrium, a subset of CTBs differentiates into extravillous trophoblasts (EVTs, expressing HLA-G, ADAM8, and PRDM5). These invasive EVTs penetrate the endometrial stroma, ultimately giving rise to trophoblastic cell columns and anchoring villi during later developmental stages. **(b)** Comparing the expression of CTB-related genes between M16 and euploid embryos. **(c)** Comparing the expression of STB-related genes between T16 and euploid embryos. **(d)** The pseudotime analysis of the integrated data with *Xiang et al*’s data^1^.


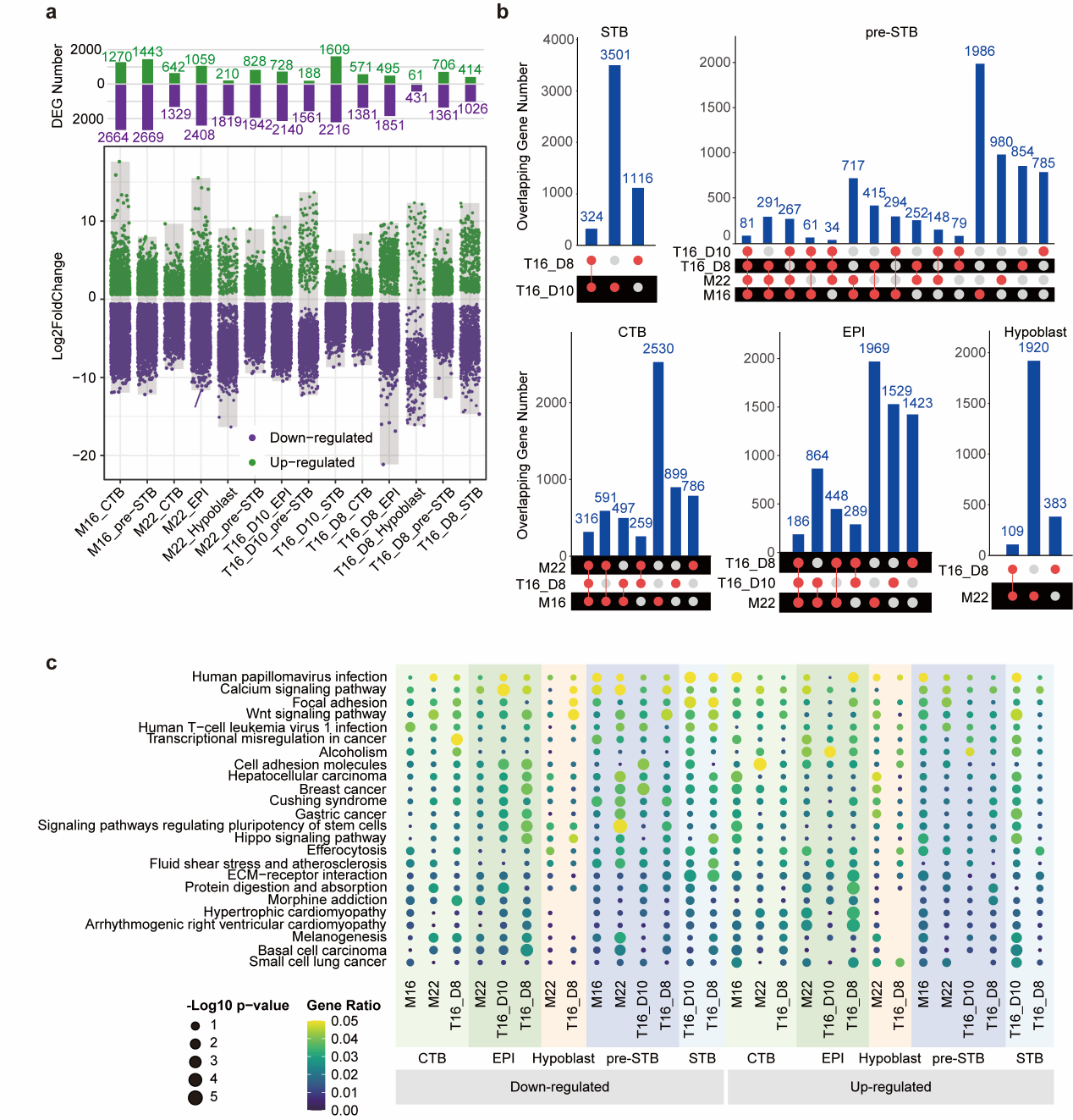


**Supplementary Figure 4**. The transcription characteristics of aneuploid embryos within lineage subtypes. For T16 embryos, analyses were conducted for both D10 and D8 stages. The M16 and M22 embryos were exclusively assessed at the D8 stage. **(a)** Differential expression analysis was conducted within specific lineage subtypes between aneuploidy and the corresponding euploid subtype. The number (top panel) and fold change (bottom panel) of DEGs were displayed. **(b)** The overlap of DEGs among karyotypes within each lineage subtype. **(c)** KEGG enrichment analysis was performed on lineage subtypes of each karyotype, showcasing significantly enriched pathways in more than six groups.


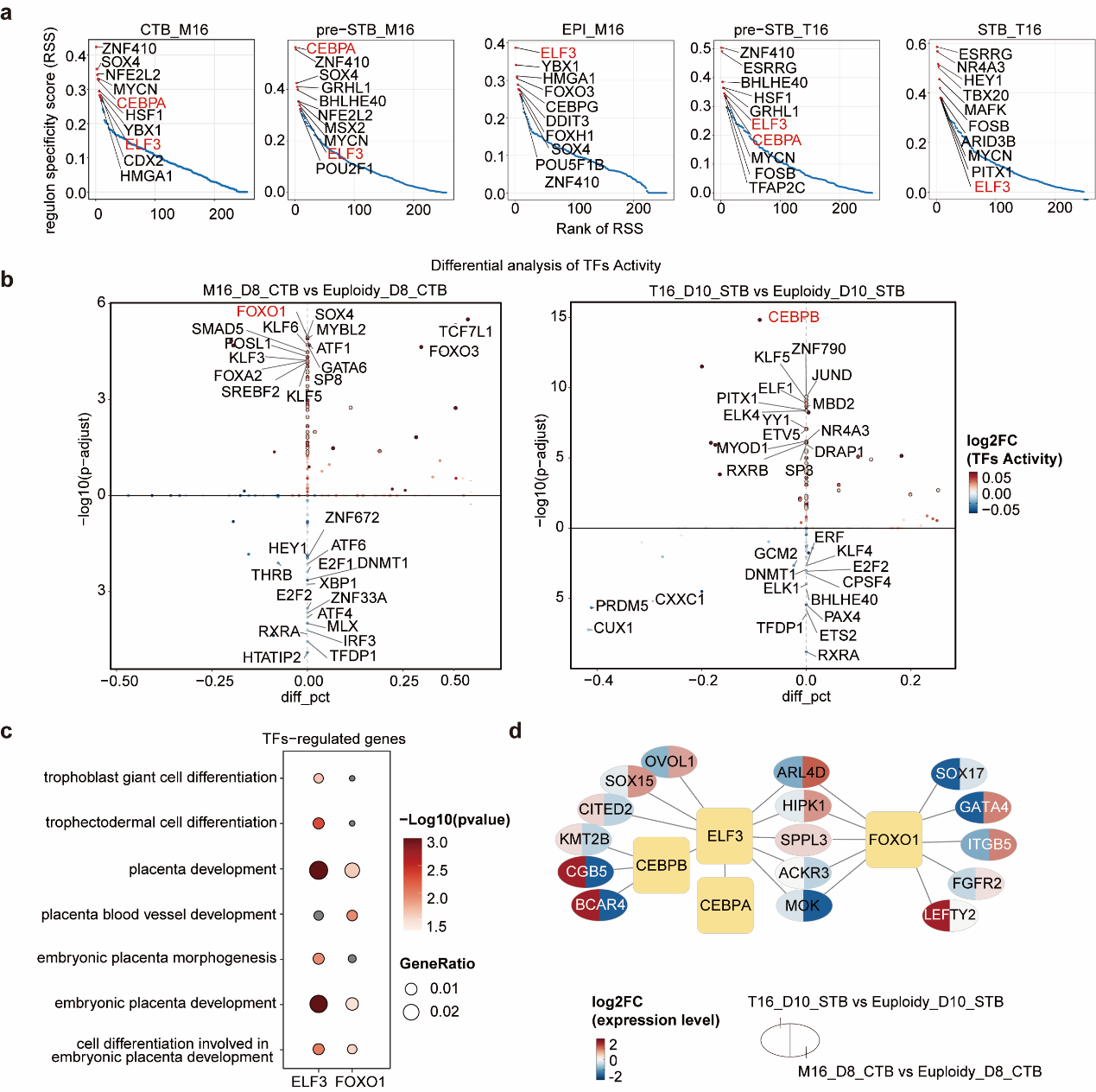


**Supplementary Figure 5**. The regulatory TFs network analysis through SCENIC. **(a)** The top 10 RSS ranking TFs for representative aneuploid subtypes. **(b)** Differential TFs activity analysis between each aneuploid subtype and its corresponding euploids, with TFs of interest highlighted in red. **(c)** The target genes of transcription factor ELF3 and FOXO1 were enriched in pathways related to placental development. **(d)** The regulatory network among transcription factor ELF3, FOXO1, CEBPA, and CEBPB. Their target genes' fold change in T16_D10_STB and M16_D8_CTB mostly exhibit opposite trends.


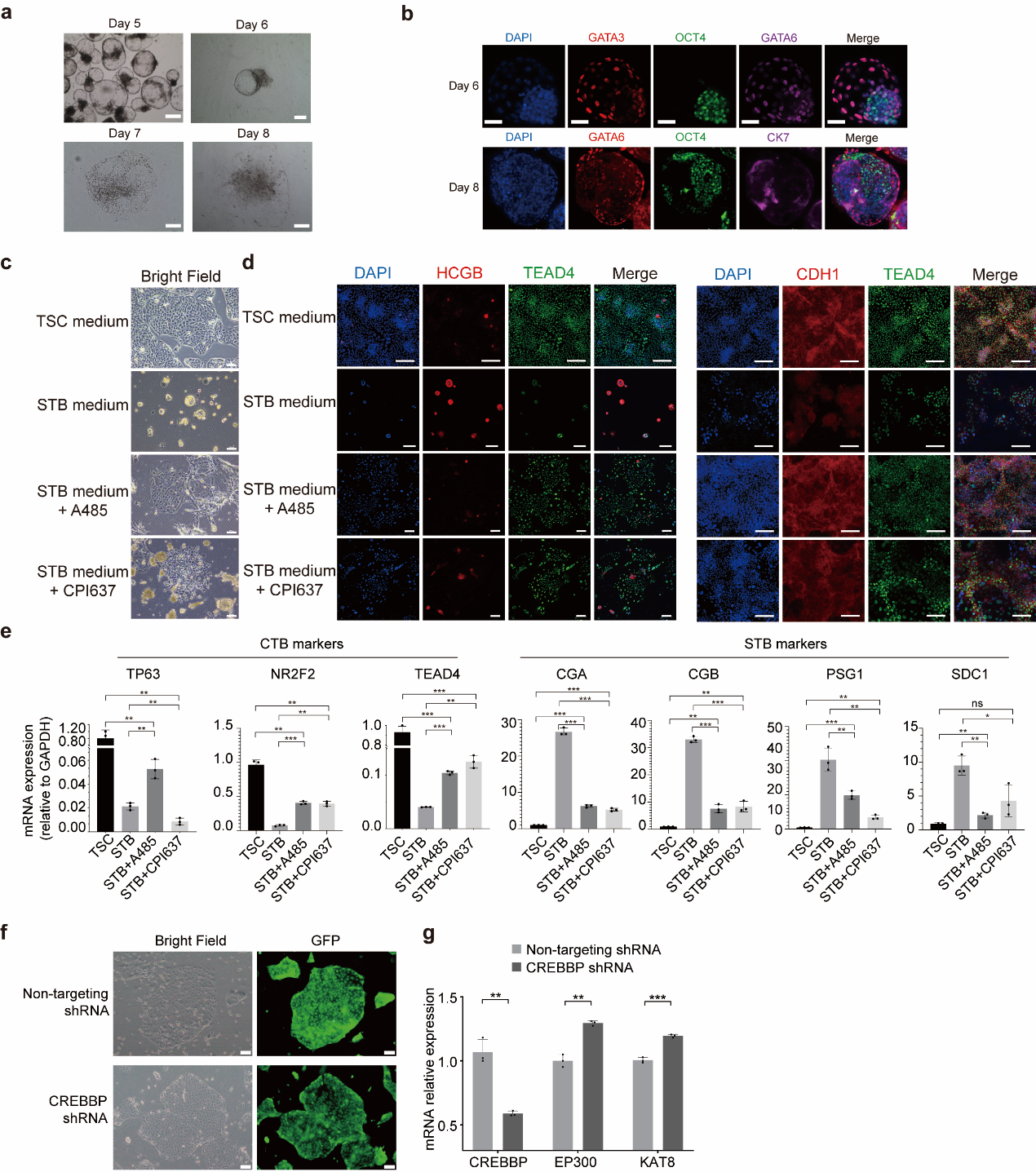


**Supplementary Figure 6**. The blastoid and TSCs models validated that *CREBBP* played a crucial role in STB development. **(a)** The bright field images showed that the blastoids adhered to the stromal cell and developed into peri-implantation embryo-like structures in IVC medium. Scale bars: 50 μm. **(b)** The immunofluorescence images showed that the epiblast surrounding to form an amnion-like cyst on day 8. Scale bars: 50 μm. **(c-e)** TSCs were induced to differentiate into STBs in groups concurrently treated with the CREBBP/EP300 inhibitors A485 and CPI637. The status of cell differentiation was observed after 5 days. **(c)** Representative bright field image of TSCs after treatment for 5 days. **(d)** Immunofluorescence microscopy images of cells stained for TEAD4 (TSC marker, gray), hCGB (STB marker, red), and DAPI (nuclei, blue) (scale bars: 200 μm). **(e)** qPCR analysis of mRNA expression in TSCs. Bars represent the mean log2-fold change (FC) ± SD relative to TSC medium (n = 3 independent experiments). **P* < 0.05, ***P* < 0.005, ****P* < 0.0005. **(f)** GFP fluorescence in the cell clones confirmed successful viral transduction. Scale bars: 100 μm. **(g)** CREBBP, EP300 and KAT8 mRNA expression relative to the non-targeting shRNA condition, measured by qPCR. Bars represent mean fold-change (FC) ± SD. **P < 0.005, ***P < 0.0005.

## Reference

1. Xiang L, Yin Y, Zheng Y, et al. A developmental landscape of 3D-cultured human pre-gastrulation embryos. *Nature*. Published online December 12, 2019. doi:10.1038/s41586-019-1875-y

2. Yu X, Liang S, Chen M, et al. Recapitulating early human development with 8C-like cells. *Cell Rep*. 2022;39(12):110994. doi:10.1016/j.celrep.2022.110994

3. Picelli S, Faridani OR, Björklund AK, Winberg G, Sagasser S, Sandberg R. Full-length RNA-seq from single cells using Smart-seq2. *Nat Protoc*. 2014;9(1):171-181. doi:10.1038/nprot.2014.006

4. Ewels P, Magnusson M, Lundin S, Käller M. MultiQC: summarize analysis results for multiple tools and samples in a single report. *Bioinforma Oxf Engl*. 2016;32(19):3047-3048. doi:10.1093/bioinformatics/btw354

5. Dobin A, Davis CA, Schlesinger F, et al. STAR: ultrafast universal RNA-seq aligner. *Bioinforma Oxf Engl*. 2013;29(1):15-21. doi:10.1093/bioinformatics/bts635

6. Liao Y, Smyth GK, Shi W. featureCounts: an efficient general purpose program for assigning sequence reads to genomic features. *Bioinforma Oxf Engl*. 2014;30(7):923-930. doi:10.1093/bioinformatics/btt656

7. Stuart T, Butler A, Hoffman P, et al. Comprehensive Integration of Single-Cell Data. *Cell*. 2019;177(7):1888-1902.e21. doi:10.1016/j.cell.2019.05.031

8. Robinson MD, McCarthy DJ, Smyth GK. edgeR: a Bioconductor package for differential expression analysis of digital gene expression data. *Bioinforma Oxf Engl*. 2010;26(1):139-140. doi:10.1093/bioinformatics/btp616

9. Yu G, Wang LG, Han Y, He QY. clusterProfiler: an R package for comparing biological themes among gene clusters. *Omics J Integr Biol*. 2012;16(5):284-287. doi:10.1089/omi.2011.0118

10. Korsunsky I, Millard N, Fan J, et al. Fast, sensitive and accurate integration of single-cell data with Harmony. *Nat Methods*. 2019;16(12):1289-1296. doi:10.1038/s41592-019-0619-0

11. Smirnov P, Safikhani Z, El-Hachem N, et al. PharmacoGx: an R package for analysis of large pharmacogenomic datasets. *Bioinforma Oxf Engl*. 2016;32(8):1244-1246. doi:10.1093/bioinformatics/btv723

12. Gu Z, Eils R, Schlesner M. Complex heatmaps reveal patterns and correlations in multidimensional genomic data. *Bioinforma Oxf Engl*. 2016;32(18):2847-2849. doi:10.1093/bioinformatics/btw313

13. Trapnell C, Cacchiarelli D, Grimsby J, et al. The dynamics and regulators of cell fate decisions are revealed by pseudotemporal ordering of single cells. *Nat Biotechnol*. 2014;32(4):381-386. doi:10.1038/nbt.2859

14. Aibar S, González-Blas CB, Moerman T, et al. SCENIC: single-cell regulatory network inference and clustering. *Nat Methods*. 2017;14(11):1083-1086. doi:10.1038/nmeth.4463

15. Shannon P, Markiel A, Ozier O, et al. Cytoscape: a software environment for integrated models of biomolecular interaction networks. *Genome Res*. 2003;13(11):2498-2504. doi:10.1101/gr.1239303

16. Chen T, Chen X, Zhang S, et al. The Genome Sequence Archive Family: Toward Explosive Data Growth and Diverse Data Types. *Genomics Proteomics Bioinformatics*. 2021;19(4):578-583. doi:10.1016/j.gpb.2021.08.001

17. CNCB-NGDC Members and Partners. Database Resources of the National Genomics Data Center, China National Center for Bioinformation in 2022. *Nucleic Acids Res*. 2022;50(D1):D27-D38. doi:10.1093/nar/gkab951

18. Rossant J. Human embryology: Implantation barrier overcome. *Nature*. 2016;533(7602):182-183. doi:10.1038/nature17894

19. Kuna M, Dhakal P, Iqbal K, et al. CITED2 is a conserved regulator of the uterine-placental interface. *Proc Natl Acad Sci U S A*. 2023;120(3):e2213622120. doi:10.1073/pnas.2213622120

20. Imakawa K, Dhakal P, Kubota K, et al. CITED2 modulation of trophoblast cell differentiation: insights from global transcriptome analysis. *Reprod Camb Engl*. 2016;151(5):509-516. doi:10.1530/REP-15-0555

21. Renaud SJ, Chakraborty D, Mason CW, Rumi MAK, Vivian JL, Soares MJ. OVO-like 1 regulates progenitor cell fate in human trophoblast development. *Proc Natl Acad Sci U S A*. 2015;112(45):E6175-6184. doi:10.1073/pnas.1507397112

22. Kovács KA, Steinmann M, Magistretti PJ, Halfon O, Cardinaux JR. CCAAT/Enhancer-binding Protein Family Members Recruit the Coactivator CREB-binding Protein and Trigger Its Phosphorylation *. *J Biol Chem*. 2003;278(38):36959-36965. doi:10.1074/jbc.M303147200

23. Jurado LA, Song S, Roesler WJ, Park EA. Conserved amino acids within CCAAT enhancer-binding proteins (C/EBP(alpha) and beta) regulate phosphoenolpyruvate carboxykinase (PEPCK) gene expression. *J Biol Chem*. 2002;277(31):27606-27612. doi:10.1074/jbc.M201429200

24. Duong DT, Waltner-Law ME, Sears R, Sealy L, Granner DK. Insulin inhibits hepatocellular glucose production by utilizing liver-enriched transcriptional inhibitory protein to disrupt the association of CREB-binding protein and RNA polymerase II with the phosphoenolpyruvate carboxykinase gene promoter. *J Biol Chem*. 2002;277(35):32234-32242. doi:10.1074/jbc.M204873200

25. Schaufele F, Enwright JF, Wang X, et al. CCAAT/enhancer binding protein alpha assembles essential cooperating factors in common subnuclear domains. *Mol Endocrinol Baltim Md*. 2001;15(10):1665-1676. doi:10.1210/mend.15.10.0716

26. Guo S, Cichy SB, He X, et al. Insulin suppresses transactivation by CAAT/enhancer-binding proteins beta (C/EBPbeta). Signaling to p300/CREB-binding protein by protein kinase B disrupts interaction with the major activation domain of C/EBPbeta. *J Biol Chem*. 2001;276(11):8516-8523. doi:10.1074/jbc.M008542200

27. Erickson RL, Hemati N, Ross SE, MacDougald OA. p300 coactivates the adipogenic transcription factor CCAAT/enhancer-binding protein alpha. *J Biol Chem*. 2001;276(19):16348-16355. doi:10.1074/jbc.m100128200

28. Wang H, Fang R, Cho JY, Libermann TA, Oettgen P. Positive and Negative Modulation of the Transcriptional Activity of the ETS Factor ESE-1 through Interaction with p300, CREB-binding Protein, and Ku 70/86 *. *J Biol Chem*. 2004;279(24):25241-25250. doi:10.1074/jbc.M401356200

29. Van Der Heide LP, Smidt MP. Regulation of FoxO activity by CBP/p300-mediated acetylation. *Trends Biochem Sci*. 2005;30(2):81-86. doi:10.1016/j.tibs.2004.12.002

30. Matsuzaki H, Daitoku H, Hatta M, Aoyama H, Yoshimochi K, Fukamizu A. Acetylation of Foxo1 alters its DNA-binding ability and sensitivity to phosphorylation. *Proc Natl Acad Sci*. 2005;102(32):11278-11283. doi:10.1073/pnas.0502738102

31. Van Voorden AJ, Keijser R, Veenboer GJM, et al. EP300 facilitates human trophoblast stem cell differentiation. *Proc Natl Acad Sci*. 2023;120(28):e2217405120. doi:10.1073/pnas.2217405120
